# Enhanced Glucose Uptake in Human Liver Cells and Inhibition of Carbohydrate Hydrolyzing Enzymes by Nordic Berry Extracts

**DOI:** 10.3390/molecules22101806

**Published:** 2017-10-24

**Authors:** Giang Thanh Thi Ho, Thi Kim Yen Nguyen, Eili Tranheim Kase, Margey Tadesse, Hilde Barsett, Helle Wangensteen

**Affiliations:** 1 Department of Pharmaceutical Chemistry, School of Pharmacy, University of Oslo, P.O. Box 1068 Blindern, 0316 Oslo, Norway; anna_2391@hotmail.com (T.K.Y.N.); margey.tadesse@farmasi.uio.no (M.T.); hilde.barsett@farmasi.uio.no (H.B.); helle.wangensteen@farmasi.uio.no (H.W.); 2 Department of Pharmaceutical Biosciences, School of Pharmacy, University of Oslo, P.O. Box 1068 Blindern, 0316 Oslo, Norway; e.t.kase@farmasi.uio.no

**Keywords:** 15-lipoxygenase, xanthine oxidase, α-glucosidase, α-amylase, polyphenols, glucose uptake

## Abstract

A Western lifestyle with low physical activity and a diet rich in sugar, fat and processed food contribute to higher incidences of diabetes and obesity. Enhanced glucose uptake in human liver cells was observed after treatment with phenolic extracts from different Nordic berries. All berry extracts showed higher inhibition against α-amylase and α-glucosidase than the anti-diabetic agent acarbose. Total phenolic content and phenolic profiles in addition to antioxidant activities, were also investigated. The berries were extracted with 80% methanol on an accelerated solvent extraction system (ASE) and then purified by C-18 solid phase extraction (SPE). Among the ASE methanol extracts, black chokeberry, crowberry and elderberry extracts showed high stimulation of glucose uptake in HepG2 cells and also considerable inhibitory effect towards carbohydrate hydrolyzing enzymes. SPE extracts with higher concentrations of phenolics, resulted in increased glucose uptake and enhanced inhibition of α-amylase and α-glucosidase compared to the ASE extracts. Crowberry and cloudberry were the most potent 15-lipoxygenase inhibitors, while bog whortleberry and lingonberry were the most active xanthine oxidase inhibitors. These results increase the value of these berries as a component of a healthy Nordic diet and have a potential benefit against diabetes.

## 1. Introduction

The increasing incidence of type 2 diabetes (T2D) throughout the world is closely linked to westernized dietary patterns, physical inactivity and rising rates of obesity [1]. Dietary intervention seems to be a reasonable option in the treatment of T2D. The Nordic diet, characterized by high consumption of oily fish, vegetables, berries and whole grains, has in recent years gained increased attention due to its potential to reduce the risk for diseases associated with metabolic syndrome such as T2D [2,3]. Berries are a good source of phenolics such as phenolic acids, flavonols, flavanols, anthocyanins, proanthocyanidins and ellagitannins, and the high content of these phenolics is associated with increased antioxidant effects. Berries are also rich in vitamins, minerals and dietary fiber [4]. In epidemiological and clinical studies, a high intake of berries has been associated with improved cardiovascular risk profiles, significant decrease in low-density lipoprotein (LDL) oxidation and lipid peroxidation, increased total plasma antioxidant capacity, less dyslipidemia and improved glucose utilization [4,5].

The liver plays an important role in blood glucose control, storage and utilization of glucose. It is responsible for providing glucose to both insulin insensitive (nervous, skin, red blood cells, smooth muscle, etc.) and insulin sensitive (skeletal muscle and fat) tissues [6]. In view of the central role of the liver in maintaining glucose homeostasis, substances which stimulate glucose uptake in the liver might play an important role in the pathogenesis of T2D. The key enzymes which catalyze the final step in the digestive process of carbohydrates in mammals are α-amylase and α-glucosidase. Hence, α-amylase and α-glucosidase inhibitors can delay glucose absorption, resulting in reduced postprandial plasma glucose levels and suppressed postprandial hyperglycemia [7]. There is some evidence that phenolics from berries can inhibit α-amylase and α-glucosidase, so consumption of berry phenolics can inhibit these enzymes locally in the small intestine without the need for absorption [8]. Free radicals and oxidative stress are involved in a number of disease states such as cancer, atherosclerosis and diabetes [9]. In diabetes, 15-lipoxygenase (15-LO) and xanthine oxidase (XO) are involved in free radical production [9]. For this reason, intake of antioxidants and inhibitors of 15-LO and XO has emerged as additional attractive therapeutic strategies to combat T2D [10].

In this study, 14 wild or cultivated Nordic berries (bilberry, blackberry, black chokeberry, black currant, blueberry, bog whortleberry, cloudberry, crowberry, elderberry, lingonberry, raspberry, red currant, rowanberries and sea buckthorn) were extracted with 80% methanol using an accelerated solvent extraction system (ASE) and then purified by C-18 solid phase extraction (SPE). The aim was to investigate the glucose uptake in human liver cells and the inhibition of the enzymes α-amylase, α-glucosidase, 15-LO and XO after exposure to the berry ASE and SPE extracts. The content of the main phenolics of the active extracts were characterized as well.

## 2. Results and Discussion

### 2.1. Total Phenol Content and Phenolic Profile of Extracts

Hexane extraction was performed prior to extraction with 80% methanol to remove waxes and other highly lipophilic substances from the berry materials. The yield of the hexane extracts gave a median value of 1.0% of the dried berries. Then 80% aqueous methanol was used since aqueous alcohol is a good solvent for the extraction of phenolics [11]. Table 1 shows the phenolic content of all berry extracts.

Crowberries, chokeberries and bog whortleberries were found to have the highest content of phenolics both in the methanol ASE extract and in the fresh berries. Purification by SPE resulted in extracts that contained from 5 to 25 times (average 8.8) more total phenolics compared to the ASE extracts. In addition to crowberry, chokeberry and bog whortleberry, the cloudberry methanol SPE extract also showed a high phenolic content. The results are comparable with literature values [12]. The Folin–Ciocalteu reagent was used for quantifying the phenolic content in the berry extracts. The presence of other easily oxidizable substances, which are not phenolic compounds, such as sugars, ascorbic acid and vitamins may also react with the Folin–Ciocalteu reagent, causing an overestimation of total phenolic content [13], particularly in the ASE extracts before SPE purification. This study shows a pronounced difference in phenolic content between wild bilberries (*Vaccinium myrtillus*) (533 mg gallic acid equivalents (GAE)/100 g fresh weight) and the cultivated blueberries (*Vaccinium corymbosum*) (211 mg GAE/100 g). *V. corymbosum* do not have a long tradition in the Nordic diet but is gaining increased popularity among consumers.

NMR spectroscopy was employed to identify the main phenolic constituents of the extracts, together with data of the already known major phenolics of the berries. An advantage with ^1^H-NMR is that organic molecules containing hydrogen atoms will give signals in the spectrum at positions dependent on the surroundings of each hydrogen atom, and the area of the signals depends on the number of atoms giving rise to each signal and makes it possible to detect e.g., anthocyanins, flavonols, organic acids and sugars in the same spectrum. NMR spectroscopy also has the advantage of being a reproducible, non-destructive, robust and fast method, with no need for chromatographic separation ahead of the analysis [14]. The methanol ASE extracts were dominated by signals from carbohydrates (δ 3–4 ppm, anomeric proton doublets δ 4.4–5.4 ppm) and signals from organic acids such as citric acid and malic acid (δ 2.6–2.9 ppm). The ^1^H-NMR spectra of the SPE methanol extracts showed that the intensity of the sugar signals was much reduced comparative to other signals in the spectra, an indication that SPE purification had removed free sugars, resulting in extracts enriched in aromatic compounds. The major phenolic constituents in each SPE extract were identified by comparison with NMR literature values. The presence of the phenolic compounds in each berry extract was further confirmed by assessment of the literature (Table 2). A large number of published works describe the content of anthocyanins and other types of flavonoids and phenolics in the berries used [15,16,17,18,19,20,21].

Anthocyanins were predominant in the dark coloured berry extracts and the aglycones were identified mainly based on the B-ring signals [40,41]. Cyanidins were identified by their H-6’ dd signals at δ 8.2–8.3 ppm and H-2’ doublet at δ 8.0–8.1 ppm, respectively, delphinidins by their H-2’ and H-6’ singlet at δ 7.7–7.8 ppm and malvidins with an H-2’ and H-6’ singlet at δ 7.9 ppm. The number of signals at δ ca. 9.0 ppm attributed to H-4 indicated the number of anthocyanins present in each extract, and the shift value at δ ca. 9.0 ppm indicated that anthocyanins were present in the flavylium form, as well. A few berry extracts (bog whortleberry, elderberry and sea buckthorn) were rich in flavonol glycosides. Here, too, the B-ring signals were used to distinguish between the aglycones [42]. Quercetin, myricetin and isorhamnetin glycosides give meta-coupled doublets with the H-6 and H-8 signals (A-ring protons) at ca. δ 6.2 and 6.4 ppm and with a coupling constant of 2 Hz. The B-ring signals differed: quercetin (7.7–7.8 ppm, d, *J* = 2 Hz, H-2’; 6.8 ppm, d, *J* = 8 Hz, H-5’; 7.6 ppm, dd, *J* = 8, 2 Hz, H-6’), myrcetin (7.3–7.4 ppm, s, H-2’ and H-6’) and isorhamnetin (7.9 ppm, d, *J* = 2 Hz, H-2’; 6.9 ppm, d, *J* = 8 Hz, H-5’; 7.6 ppm, dd, *J* = 8, 2 Hz, H-6’; 3.95 ppm, s, -OCH3). Chlorogenic acids, characterized by the olefinic proton signals in the caffeoyl moiety at ca. 6.3 and ca. 7.6 ppm with a coupling constant of 15–16 Hz and quinic acid signals at 1.9–2.2 ppm [43], were present in high amounts in the rowanberry extract, and somewhat less in black chokeberry, blueberry and elderberry extracts. Cloudberry and raspberry extracts contained high levels of ellagitannins, characterized by the singlets (δ 6.3–6.7 ppm) from protons on the hexahydroxydiphenoyl (HHDP) moieties and highly shielded anomeric proton signals [44]. Benzoic acids were characterized by their deshielded doublet signals at δ 8.0–8.1 ppm (1H), and two sets of triplets at δ 7.5–7.6 (1H) and 7.4–7.5 ppm (2H) [35], and were identified in the cloudberry and lingonberry SPE extracts. The ^1^H-NMR spectra of the SPE berry extracts are presented in the supplementary materials.

**Bilberry SPE Extract**: Ten different signals from H-4 in anthocyanins were observed, four were major. This variety in content of anthocyanins gave rise to a complex spectrum with difficulties in identifying specific anthocyanins. However, signals from cyanidin and delphinidin dominated, followed by malvidin, in accordance with the literature [22,23].

**Blackberry SPE Extract**: There were two sets of signals from anthocyanins, with cyanidin 3-glucoside as the major one. One rhamnose methyl signal (δ 1.15, *J* = 6 Hz) indicated the presence of cyanidin 3-rutinoside, which is in accordance with the literature [24,25].

**Black chokeberry SPE Extract**: Two major anthocyanins were present, cyanidin 3-galactoside and cyanidin 3-arabinoside, which were previously reported to be the major glycosides. In addition, signals from both chlorogenic acid and neochlorogenic acid were prominent [19,21,26].

**Black currant SPE Extract**: Signals from two main anthocyanins were observed: delphinidin (major) and cyanidin types. Two sets of signals from the methyl group in rhamnose indicated the presence of delphinidin-3-rutinoside and cyaniding-3-rutinoside [19,21,27].

**Blueberry SPE Extract**: Similar to the bilberry extract, blueberries gave rise to a crowded spectrum of anthocyanin signals, 15 signals from H-4 in the anthocyanin could be observed. There were no major signals from cyaniding or peonidin (H-6’, dd, δ 8.2–8.3 ppm), and malvidin and delphinidin type anthocyanins were the dominating ones. Chlorogenic acid was the major non-flavonoid present [21,27,28].

**Bog whortleberry SPE Extract**: The spectrum was dominated by signals from glycosides of the four main aglycones: quercetin, myricetin, malvidin and delphinidin. The H-4 signal from anthocyanins indicated the presence of at least seven anthocyanins, from the B-ring signals maldivin seemed to be the major aglycone (δ 7.92 (s)), which could be due to malvidin-3-glucoside or malvidin-3-galactoside, previously reported to be the major aglycones [27,29,30].

**Cloudberry SPE Extract**: There were signals from two major compounds, benzoic acid and an ellagitannin. The spectrum contained six characteristic singlets from protons in the HHDP units (δ 6.3–6.7 ppm) of the ellagitannin, a broad singlet at δ 7.02 ppm corresponding to protons in galloyl units of the molecule, and two shielded anomeric proton signals (δ 5.98, d, *J* = 8.7 Hz and δ 6.40, d, *J* = 4.0 Hz). This is consistent with the literature values for sanguiin H6, a major ellagitannin in cloudberries [31,32]. Benzoic acid signals were also observed.

**Crowberry SPE Extract**: Signals from three major anthocyanins were prominent, they were of delphinidin, malvidin and cyanidin type. Plausible identities are delphinidin-3-galactoside, cyanidin-3-galactoside and malvidin-3-galactoside, according to the literature [21,27,33].

**Elderberry SPE extract**: The spectrum showed the presence of four major compounds, cyanidin-3-glucoside, cyanidin-3-sambubioside, rutin and chlorogenic acid, which is in accordance with the literature [19,27,34].

**Lingonberry SPE Extract**: Signals from benzoic acids were the most prominent in the aromatic region, identified as 1-*O*-benzoyl-glucose and 6-*O*-benzoyl-glucose. Both α (*J* = 3.5 Hz) and β (*J* = 7.5 Hz) anomeric proton signals were present. Minor cyanidin signals were observed [21,27,35].

**Raspberry SPE Extract**: The ^1^H-NMR signals matched with the ellagitannin signals from cloudberries, thus indicating that the major aromatic compound was sanguiin H-6, which is in accordance with a recent review. Minor cyanidin signals were observed [31,36,37].

**Red currant SPE Extract**: Weak signals in the aromatic area were observed with no dominating phenolics. The aromatic signals were possibly glycosylated phenolic acids, which have previously been reported in these berries [19]. Traces of cyanidin signals were observed [19,21].

**Rowanberry SPE Extract**: The major aromatic compounds were chlorogenic acid and neochlorogenic acid, present in an approximately 1:1 ratio [21,38].

**Sea buckthorn SPE Extract**: Two sets of isorhamnetin signals dominated the aromatic region of the NMR spectrum. The presence of signals from rhamnose methyl groups at δ 0.9–1.0 ppm indicate isorhamnetin rhamnosides which are common in this species [21,27,39].

### 2.2. Glucose Uptake in HepG2-Cells

**Figure 1:**
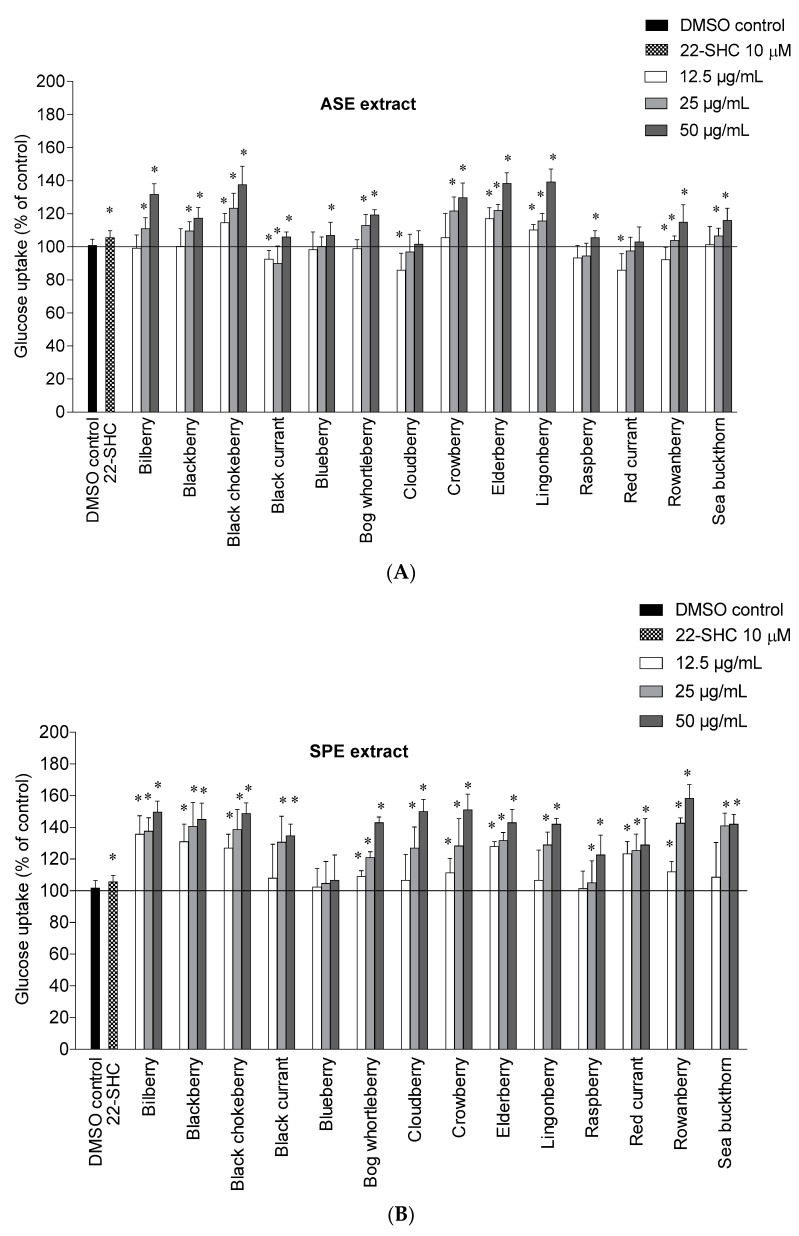
Effects of berry extracts on glucose uptake in human liver cells. HepG2-cells were treated with (**A**) 12.5, 25 and 50 µg/mL of different berry ASE extracts and (**B**) 12.5, 25 and 50 µg/mL of different berry SPE extracts for 24 h. Thereafter, the cells were exposed to d-[^14^C(U)]glucose (1 μCi/mL, 100 μM) for 4 h as described in Materials and Methods. 22-*S*-hydroxycholesterol (22-SHC) (10 μM) was used as positive control. The figures show d-[^14^C(U)]glucose uptake given as means ± SEM (*n* = 3) from separate experiments. * *p* < 0.05 vs. control (0.1% DMSO).

This study shows for the first time the potential of Nordic berry extracts to stimulate glucose uptake in human liver cells. The ASE and SPE extracts were tested at 12.5, 25 and 50 µg/mL and the results are shown in Figure 1A,B. The cytotoxicity of the berry extracts on the liver cells was determined by the Bradford protein method [45]. In this case none of the berry extracts showed a drastic change of protein levels in the cells and were therefore not cytotoxic to the liver cells.

Among the ASE extracts, lingonberry and elderberry showed the highest stimulation of uptake at 50 µg/mL, with an increase of 39.3 ± 7.6% and 38.3 ± 6.5% respectively, followed by black chokeberry (37.7 ± 11.0%), bilberry (31.7 ± 6.5%) and crowberry (29.7 ± 9.0%) compared to 0.1% DMSO control. The bilberry, blackberry, bog whortleberry, crowberry, rowanberry and sea buckthorn ASE extracts showed a significant increase only at 25 µg/mL and 50 µg/mL, while the black currant, blueberry and raspberry ASE extracts only showed a significant increase at 50 µg/mL. For all berries, the glucose uptake was higher with the SPE extracts compared to the ASE extracts. It seems that higher content of polyphenols and simpler phenolics are important for the mode of action.

A high content of anthocyanins seems to be important for glucose uptake in several of the berries. The anthocyanin rich extracts of bilberry, blackberry, black chokeberry, bog whortleberry, crowberry and elderberry significantly increased the glucose uptake at 25 and 50 µg/mL for the ASE extracts and at all tested concentrations for the SPE extracts. The importance of anthocyanins is supported by other studies showing that anthocyanins can stimulate the glucose uptake in liver and skeletal muscle cells [46,47]. The rowanberry SPE extract showed the highest maximal efficacy on glucose uptake at 50 µg/mL with an increase of 58.3 ± 8.5%, followed by the crowberry SPE extract (51.0 ± 9.8%), the cloudberry SPE extract (50.0 ± 7.5%), and the bilberry SPE extract (49.7 ± 6.8%). There does not seem to be one single constituent that contributes to the effect, rather a number of phenolic substances can stimulate the uptake as we have recently described [46,48]. Chlorogenic acids likely play an important role in the uptake of glucose observed for rowanberry, and might also be involved in the high activity observed for black chokeberry and elderberry in addition to cyanidin glycosides [48]. Ellagitannins present in cloudberry and raspberry (Table 2), and cyanidin glycosides present in crowberry might be important contributors in these berries.

Benzoic acid, which is a major constituent of the cloudberry SPE extract, has in a previous study shown a small increase of glucose uptake in human myotubes at 10 µM [48]. The bilberry SPE extract contains a complex mixture of delphinidin-, cyanidin- and malvidin glycosides, and their individual contribution to the glucose uptake is difficult to predict. There is a need for more studies to investigate how the different anthocyanin aglycones contribute to this effect. The SPE extracts from blackberry, black chokeberry, bog whortleberry, elderberry, lingonberry and sea buckthorn showed a significant increase of glucose uptake in the range of 42.0–48.7% at the highest tested concentration (50 µg/mL) compared to DMSO control. At the lowest concentration (12.5 µg/mL) the anthocyanin rich extracts bilberry SPE extract, the blackberry SPE extract and the elderberry SPE extract were the most potent with increased glucose uptake of 35.7 ± 11.6%, 31.0 ± 11.0% and 28.0 ± 3.0%, respectively. Quercetin and isorhamnetin glycosides from the flavonol-rich extracts from elderberry, sea buckthorn and bog whortleberry might also contribute to the glucose stimulation [48]. The high content of benzoyl glycosides in lingonberry and phenolic acids in red currant might also play a major role in the uptake of glucose in HepG2-cells. The sea buckthorn SPE extract showed the lowest phenolic content (Table 1), but still showed a high increase of glucose uptake compared to the other SPE extracts. It seems that the type of phenolic is more important for the uptake of glucose than the total phenolic content. In view of the potent effect of cloudberry and lingonberry SPE extracts rich in benzoic acids or its derivatives, benzoic acid derivatives should be subjects for further studies, in addition to isolated anthocyanins and flavonols. The enhanced uptake of glucose observed for these berry extracts in the liver cells might be effective in the skeletal muscle cells as well. We have previously seen that the stimulation of glucose uptake by the phenolics is similar in both the liver cells and the skeletal muscle cells [48].

### 2.3. Inhibition of α-Amylase and α-Glucosidase

Small intestinal α-glucosidase and pancreatic α-amylase are key enzymes of dietary carbohydrate digestion in humans. Inhibitors of these enzymes by phenolic rich extracts may offer a natural dietary approach to prevent T2D because it will be effective in retarding carbohydrate digestion and glucose absorption to suppress postprandial hyperglycemia. The ASE extracts of the 14 Nordic berries were all potent inhibitors of α-amylase with IC_50_ values ranging from 6.3 to 21.4 µg/mL (Table 3).

Among the ASE extracts, the crowberry (IC_50_ 6.3 ± 0.8 µg/mL) and the bog whortleberry (IC_50_ 9.1 ± 3.5 µg/mL) showed the highest α-amylase activity, followed by cloudberry (IC_50_ 9.6 ± 0.7 µg/mL). The SPE extracts appeared to have equal or somewhat higher α-amylase inhibitory activity compared to ASE extracts, with the anthocyanin-rich crowberry (5.3 ± 0.9 µg/mL) and black chokeberry (6.0 ± 1.0 µg/mL) extracts being the most active ones. Regarding the α-glucosidase inhibitory activity of the SPE extracts, the crowberry, the chokeberry and the cloudberry possessed the highest inhibitory activity, with IC_50_ values of 10.9 ± 1.1, 12.0 ± 1.7 and 13.6 ± 1.3 µg/mL, respectively. All the tested ASE and SPE extracts were more potent than the acarbose used as control compound (IC_50_ 84.7 ± 3.8 µg/mL for α-glucosidase, IC_50_ 73.3 ± 4.3 µg/mL for α-amylase). Bilberry, black currant, blackberry, black chokeberry, red currant and lingonberry have been reported to be potent α-amylase inhibitors [49,50], while the inhibition of α-glucosidase has been reported for many of the berries, except for bog whortleberry, sea buckthorn and cloudberry [49,51,52]. It has been reported that berry anthocyanins and ellagitannins play an important role in the inhibition of α-amylase and α-glucosidase, respectively [8]. This study indicates that ellagitannins might contribute to a higher activity towards α-glucosidase since the raspberry and cloudberry were among the most active extracts against this enzyme. Since cloudberry and raspberry extracts contain the ellagitannin sanguiin-6, the possibility of the precipitation of enzyme–tannin complexes cannot be ruled out [53]. However, no such precipitation was observed. The other extracts appear not to contain high amounts of tannins. Furthermore, precipitation at neutral or basic pH values would not be expected [54,55]. Anthocyanin rich extracts were most active as α-glucosidase inhibitors. Rowanberry, with its high content of chlorogenic acids, was also highly active in this assay. As we have recently shown [46,48], chlorogenic and neochlorogenic acids, anthocyanins and flavonoids are potent inhibitors of α-amylase and α-glucosidase and contribute to the observed effects.

### 2.4. 15-Lipoxygenase and Xanthine Oxidase Inhibition

15-LO and XO inhibition is of interest, as these enzymes have been proposed to be involved in a number of diseases such as cancer, diabetes and cardiovascular diseases [56]. Both 15-LO and XO are thought to be involved in ROS production in diabetes [57]. The inhibitory effect of the berries towards peroxidation of linoleic acid catalyzed by soybean 15-LO and the superoxide-producing enzyme XO from cow’s milk are shown in Table 4.

The methanol ASE extracts were almost inactive as 15-LO and XO inhibitors with IC_50_ values > 167 µg/mL. Among these, the strongest inhibition of 15-LO was observed after the addition of crowberry extract with 12.3 ± 3.2% 15-LO inhibition at 83 µg/mL. Black currant ASE extract inhibited XO weakly (9.0 ± 1.9%) at 83 µg/mL. After SPE purification, the enzyme inhibition potency increased considerably (Table 4). Crowberry, cloudberry, black currant and raspberry extracts were most active against 15-LO with IC_50_ values between 46.6 and 53.5 µg/mL. Among the SPE extracts, bog whortleberry (IC_50_ 50.4 ± 1.4 µg/mL) and lingonberry (IC_50_ 67.2 ± 1.7 µg/mL) were the most active XO inhibitors. With a few exceptions [51], 15-LO and XO inhibition have, as far as we know, not been evaluated for this group of berries and show additional promising effects of the polyphenol rich berries. However, several of the polyphenols found in berries have been shown to inhibit 15-LO and XO [58,59].

## 3. Materials and Methods

### 3.1. Berries

A list of the berries used in this study is given in Table 5. They were picked at full ripeness and stored at −20 °C until extraction. Voucher specimens are kept at School of Pharmacy, University of Oslo.

### 3.2. Chemicals

Methanol-*d*_4_, tetramethylsilane, quercetin, linoleic acid, soybean 15-lipoxygenase (15-LO), hypoxanthine, xanthine oxidase (XO) from bovine milk, acarbose, 22-*S*-hydroxycholesterol (22-SHC), 4-nitrophenyl α-d-glucopyranoside (PNP-G), 2-chloro-4-nitrophenyl-α-d-maltotrioside (CNPG3), quercetin and gallic acid were from Sigma-Aldrich (St. Louis, MO, USA), Folin Ciocalteu reagent was from Merck (Darmstadt, Germany). Dulbecco‘s modified Eagle’s medium (DMEM-Glutamax™, 5.5 mM), DMEM, fetal bovine serum, Ultroser G, penicillin–streptomycin–amphotericin B and trypsin-EDTA were obtained from Gibco Life Technologies (Paisley, UK). d-[^14^C(U)]glucose (1 μCi/mL, 100 μM) was purchased from ARC (American Radiolabeled Chemicals, St. Louis, MO, USA). Corning CellBIND tissue culture plates were obtained from Corning Life-Sciences (Amsterdam, The Netherlands). The protein assay reagent was obtained from BioRad (Copenhagen, Denmark). All other reagents were of the highest purity available. All solvents were of analytical grade and water was purified by a MilliQ system (Millipore, Bedford, MA, USA).

### 3.3. Pressurized Solvent Extraction (ASE)

Frozen berries were smashed and lyophilized, then ground to a powder and extracted on an accelerated solvent extraction system, ASE 350 (Dionex, Sunnyvale, CA, USA). For extraction, 12 g berry powder was mixed with diatomaceous earth (Dionex) (4:1) and loaded in a 100 mL steel cartridge. Extraction was performed in two cycles at 60 °C, the preheating time was 5 min and static extraction per cycle 5 min. The berries were first extracted with hexane followed by 80% methanol. Solvents were removed under vacuum to give the hexane and the methanol ASE extracts.

### 3.4. Solid Phase Extraction (SPE) of Berry Extracts

The methanol ASE extracts were purified by solid phase extraction (SPE) using Strata C18-E 2 g columns (Phenomenex, Macclesfield, UK) according to McDougall, et al. [60] with some minor modifications. Briefly, the methanol ASE extract (200 mg) was dissolved in 10 mL water containing 0.2% trifluoroacetic acid (TFA), filtered through a Pall^®^ Acrodisc^®^ 32 mm syringe filter, 0.45 µM (Pall Corporation, Newquay, UK), and applied on the column equilibrated with 0.2% aqueous TFA. The column was washed with 2 × 10 mL 0.2% TFA to remove the unbound material (e.g., free sugars), then the phenolics were eluted with 2 × 10 mL methanol containing 0.2% TFA, and the methanol fraction taken to dryness in a Genevac vacuum centrifuge (Genevac, Ipswich, UK) to give the SPE methanol extract.

### 3.5. NMR Spectroscopy

^1^H-NMR spectroscopy was conducted on a Bruker AVII 400 (for ASE extracts) or a Bruker AVII 600 (for SPE extracts) instrument (Bruker, Rheinstetten, Germany). Dried berry extract (ca. 20 mg) was dissolved in methanol-*d*_4_ containing tetramethylsilane (TMS) as reference and filled in 5 mm NMR tubes. Spectra were acquired with 128 scans, at 25 °C. NMR data were processed by the MestreNova software Version 11.0 (Mestrelab Research, Santiago de Compostela, Spain).

### 3.6. Total Phenol Content

Test substances were dissolved in DMSO and the Folin–Ciocalteu assay was carried out as previously described [61]. The absorbance was measured at 765 nm on a Biochrom Libra S32 PC UV/Vis spectrophotometer (Biochrom, Cambridge, UK). A linear calibration curve for gallic acid was obtained in the range 0–25 µg/mL to calculate the amount of gallic acid equivalents (GAE) in each sample.

### 3.7. 15-Lipoxygenase (15-LO) Inhibition

Test substances were dissolved in DMSO and the assay was carried out as previously described [61], measuring the formation of conjugated dienes as increase in absorbance at 234 nm on a Biochrom Libra S32 PC UV/Vis spectrophotometer. Quercetin was used as positive control.

### 3.8. Xanthine Oxidase (XO) Inhibition

Test substances were dissolved in DMSO and the assay was carried out as previously described [62], measuring uric acid formation as increase in absorbance at 290 nm on a Biochrom Libra S32 PC UV/Vis spectrophotometer. Quercetin was used as positive control.

### 3.9. α-Glucosidase Inhibition

The α-glucosidase inhibitory activity of the extracts was carried out as recently described [48]. Baker’s yeast α-glucosidase (EC 3.2.1.20) was purchased from Sigma-Aldrich Chemie (Steinheim, Germany). Acarbose was used as positive control.

### 3.10. α-Amylase Inhibition

The α-amylase assay was performed as recently described [48]. Porcine pancreatic α-amylase (EC 3.2.1.1) was purchased from Sigma-Aldrich Chemie GmbH (Steinheim, Germany). Acarbose was used as positive control.

### 3.11. Culturing of HepG2-Cells

The human hepatoblastoma cell line HepG2 (HB-8065; ATCC, Manassas, VA, USA) was cultured in DMEM-Glutamax™ (5.5 mM glucose) supplemented with 10% fetal bovine serum, streptomycin (100 μg/mL) and penicillin (100 units/mL) at 37 °C in 5% CO_2_.

### 3.12. Glucose Uptake in HepG2-Cells

Cells were exposed to test samples dissolved in DMSO and diluted in the HepG2 cell medium and incubated for 24 h. The final DMSO concentration was 0.1% in both the DMSO control and test samples. Thereafter, cells were exposed to d-[^14^C(U)]glucose (1 μCi/mL, 100 μM) for 4 h. A 96-well UniFilter-96 GF7B microplate was mounted on top of the CellBIND plate and CO_2_ production was measured. After incubation the cells were washed twice with ice-cold phosphate buffered saline (PBS), lysed in 0.1 M NaOH, and radioactivity measured by liquid scintillation counting [63]. The protein content of each sample was determined and the glucose uptake was calculated using protein levels for standardization [45].

### 3.13. Statistics

The results from the Folin–Ciocalteu, 15-LO, XO, α-amylase and α-glucosidase assays are the averages of three parallels ± SD. Data and figures for glucose uptake are given as mean (±SEM) from *n* = number of separate experiments. At least three parallels were included in each experiment. Comparisons of different treatments were evaluated by two-tailed and paired Student’s *t*-test.

## 4. Conclusions

As far as we know, this is the first study comparing the antidiabetic potential of 14 different Nordic berries. The phenolic rich extracts from these berries have a strong effect against α-amylase and α-glucosidase and a positive influence on glucose uptake in human liver cells in vitro. The chlorogenic acid rich rowanberry SPE extract and the anthocyanin rich bilberry SPE extract showed the highest stimulation of glucose uptake in the human liver cells. The crowberry SPE extract showed the strongest inhibition of 15-LO, while the bog whortleberry SPE extract was the strongest XO inhibitor; both were rich in anthocyanins. The crowberry extract, both among the ASE and the SPE extracts, showed the strongest α-amylase and α-glucosidase inhibition.

The polyphenols present in these berries are extensively metabolized in vivo by the gut microbiota, suggesting that the accumulation of multiple phenolic metabolites is important after oral intake of berries, as well. The metabolism and bioavailability of polyphenols is complex, and to address whether it is the native polyphenols in the gut, the microbial metabolites in the gut, or absorbed metabolites that are the most important for health beneficial effects is still under debate. Synergistic effects are also plausible [64,65,66]. Therefore, it is difficult to point out which substances are the most important after consumption of these Nordic berries. Based on the presented study we can say that the phenolic compounds, either individually or combined, might be important for both enzyme inhibiting activities and for glucose uptake in liver cells, and therefore also for the potential health beneficial effects of berries.

The antidiabetic property of the berry extracts increases the nutritional value and indicates a potential as functional food against diabetes. However, long-term clinical studies that include substantial amounts of berries in an otherwise healthy diet may be required to confirm the contribution of berry consumption to the prevention of T2D and cardiovascular disease.

## Figures and Tables

**Table 1:** Total phenolic content of fresh berries and berry extracts.

Berries	Total Phenolic Content		
	Berries ^a^	Methanol ASE Extract ^b^	Methanol SPE Extract ^b^
Bilberry	533 ± 28	56 ± 3	299 ± 5
Blackberry	511 ± 32	48 ± 3	312 ± 9
Black chokeberry	835 ± 23	64 ± 2	384 ± 17
Black currant	413 ± 26	50 ± 3	306 ± 16
Blueberry	211 ± 25	18 ± 2	218 ± 3
Bog whortleberry	595 ± 37	73 ± 5	395 ± 19
Cloudberry	311 ± 13	37 ± 2	414 ± 14
Crowberry	630 ± 24	85 ± 3	467 ± 24
Elderberry	251 ± 27	53 ± 6	262 ± 7
Lingonberry	490 ± 15	38 ± 1	233 ± 5
Raspberry	201 ± 12	26 ± 2	342 ± 17
Red currant	105 ± 8	10 ± 1	244 ± 2
Rowanberry	347 ± 11	20 ± 1	150 ± 6
Sea buckthorn	95 ± 3	11 ± 0.3	89 ± 4

^a^ Expressed as mg gallic acid equivalents (GAE)/100 g fresh material; ^b^ Expressed as mg GAE/g extract.

**Table 2:** Major phenolic compounds ^a^ in berry SPE extracts determined by ^1^H-NMR.

Berries	Anthocyanin (Anthocyanidin)			Flavonol (Glycoside)			Chlorogenic Acids		Ellagi-Tannins	Other	Ref.
	Cy ^a^	Dp ^a^	Mv ^a^	Que ^a^	Myr ^a^	Isorha ^a^	CA ^a^	NCA ^a^			
Bilberry	+	+	+								[22,23]
Blackberry	+										[24,25]
Black chokeberry	+						+	+			[19,21,26]
Black currant	+	+									[19,21,27]
Blueberry		+	+				+				[21,27,28]
Bog whortleberry		+	+	+	+						[27,29,30]
Cloudberry									+	Benzoic acid	[31,32]
Crowberry	+	+	+								[21,27,33]
Elderberry	+			+			+				[19,27,34]
Lingonberry (cowberry)										1-*O*-benzoy-glucose, 6-*O*-benzoyl-glucose	[21,27,35]
Raspberry	+								+		[31,36,37]
Red currant	tr ^b^									Glycosylated phenolic acids	[19,21]
Rowanberries							+	+			[21,38]
Sea buckthorn						+					[21,27,39]

^a^ Cy, cyanidin; Dp, delphinidin; Mv, malvinidin; Que, quercetin; Myr, myricetin; Isorha, isorhamnetin; CA, 5-*O*-caffeoyl quinic acid (chlorogenic acid); NCA, 3-*O*-caffeoyl quinic acid (neochlorogenic acid); ^b^ tr, traces; +, phenolic present

**Table 3:** α-Amylase and α-glucosidase inhibition of methanol ASE extracts and SPE extracts with IC_50_ values ± SD (in µg/mL).

Berries	α-Amylase		α-Glucosidase	
	ASE Extract	SPE Extract	ASE Extract	SPE Extract
Bilberry	12.2 ± 3.1	6.2 ± 0.9	18.2 ± 0.8	9.6 ± 1.0
Blackberry	13.7 ± 0.9	10.1 ± 0.8	16.0 ± 1.1	9.5 ± 0.7
Black chokeberry	10.6 ± 1.5	6.0 ± 1.0	12.0 ± 1.7	8.1 ± 0.8
Black currant	13.5 ± 1.2	10.3 ± 2.4	24.0 ± 3.2	17.0 ± 2.3
Blueberry	16.1 ± 1.8	16.6 ± 2.0	25.2 ± 2.9	21.3 ± 1.4
Bog whortleberry	9.1 ± 3.5	8.2 ± 1.3	18.2 ± 2.7	10.5 ± 1.6
Cloudberry	9.6 ± 0.7	6.9 ± 0.8	12.6 ± 1.3	7.8 ± 0.4
Crowberry	6.3 ± 0.8	5.3 ± 0.9	10.9 ± 1.1	8.3 ± 0.4
Elderberry	10.7 ± 0.8	7.1 ± 0.8	13.5 ± 2.0	8.2 ± 0.9
Lingonberry	17.1 ± 1.2	12.0 ± 1.5	20.7 ± 3.0	17.4 ± 3.3
Raspberry	12.1 ± 1.0	10.2 ± 1.4	15.0 ± 0.5	8.4 ± 1.2
Red currant	20.7 ± 3.0	17.3 ± 0.9	31.6 ± 3.2	17.4 ± 0.7
Rowanberry	11.3 ± 0.5	7.5 ± 1.3	13.7 ± 1.8	10.0 ± 0.3
Sea buckthorn	21.4 ± 3.2	17.2 ± 1.2	34.7 ± 3.6	17.3 ± 5.6
Acarbose (positive control)	73.3 ± 4.3		84.7 ± 3.8	

**Table 4:** 15-Lipoxygenase (15-LO) and xanthine oxidase (XO) inhibition of methanol SPE extracts with IC_50_ values ± SD (in µg/mL).

Berries	15-Lipoxygenase	Xanthine Oxidase
Bilberry	69.5 ± 3.1	122.0 ± 5.1
Blackberry	74.8 ± 7.4	96.4 ± 2.4
Black chokeberry	77.5 ± 7.3	125.0 ± 4.3
Black currant	52.2 ± 3.8	80.0 ± 14.3
Blueberry	104.1 ± 3.9	73.4 ± 2.5
Bog whortleberry	63.1 ± 3.6	50.4 ± 1.4
Cloudberry	50.9 ± 2.1	101.0 ± 2.1
Crowberry	46.6 ± 1.7	76.0 ± 3.7
Elderberry	100.3 ± 5.4	>167
Lingonberry	77.0 ± 3.6	67.2 ± 1.7
Raspberry	53.5 ± 2.5	126 ±7.4
Red currant	61.6 ± 2.8	>167
Rowanberry	>167	>167
Sea buckthorn	>167	>167
Quercetin (positive control)	28.1 ± 0.5	0.7 ± 0.2

**Table 5:** Names, genus and origin of the berries used in this study.

English Name	Scientific Name	Wild or Name of Cultivar	Genus	Origin
Bilberry	*Vaccinium myrtillus* L.	Wild	*Ericaceae*	Mountain district ^a^, Norway
Blackberry	*Rubus fruticosus*	Wild	*Rosaceae*	Oslo district ^b^, Norway
Black chokeberry	*Aronia melanocarpa* (Michx.) Elliott	Cultivar Moskva	*Rosaceae*	Oslo district ^b^, Norway
Black currant	*Ribes nigrum* L.	Cultivar Ben Tron	*Grossulariaceae*	Oslo district ^b^, Norway
Blueberry	*Vaccinium corymbosum* L.	Cultivar Royal Blue	*Ericaceae*	Purchased ^c^, Marocco
Bog whortleberry	*Vaccinium uliginosum* L.	Wild	*Ericaceae*	Mountain district, Norway
Cloudberry	*Rubus chamaemorus* L.	Wild	*Rosaceae*	Mountain district, Norway
Crowberry	*Empetrum nigrum* L.	Wild	*Empetraceae*	Mountain district, Norway
Elderberry	*Sambucus nigra* L.	Cultivar Sampo	*Adoxaceae*	Vestlandet ^d^, Norway
Lingonberry (cowberry)	*Vaccinium vitis-idaea* L.	Wild	*Ericaceae*	Purchased ^e^, Sweden
Raspberry	*Rubus idaeus* L.	Wild	*Rosaceae*	Oslo district, Norway
Red currant	*Ribes rubrum* L.	Cultivar Red Dutch	*Grossulariaceae*	Oslo district, Norway
Rowanberry	*Sorbus aucuparia* L.	Wild	*Rosaceae*	Oslo district, Norway
Sea buckthorn	*Elaeagnus rhamnoides* L. A. Nelson	Unknown cultivar	*Elaeagnaceae*	Oslo district, Norway

^a^ 61–63° N, 700–1000 MASL; ^b^ 59–60° N, 0–50 m above sea level (MASL); ^c^ Imported by Bama, Norway, produced by Fresh royal. S.L. Marocco; ^d^ 61° N, 0–50 m above sea level (MASL); ^e^ Imported by Bama, Norway, packed by Mitab Skogsbær AB, Sweden.

## Supplementary Materials

Supplementary materials are available online.

## Abbreviations

15-LO	15-lipoxygenase
22-SHC	22-*S*-hydroxycholesterol
ASE	accelerated solvent extraction
DCM	Dichloromethane
DMSO	Dimethyl sulfoxide
DPPH	1,1-diphenyl-2-picrylhydrazyl
EtOH	Ethanol
MeOH	Methanol
NMR	Nuclear magnetic resonance
ROS	Reactive oxygen species
SPE	Solid phase extraction
T2D	Type 2 diabetes
TFA	Trifluoroacetic acid
TMS	Tetramethylsilane
XO	Xanthine oxidase
