# Supplementary material for: Enhanced Glucose Uptake in Human Liver Cells and Inhibition of Carbohydrate Hydrolyzing Enzymes by Nordic Berry Extracts
Source: Molecules. 2017 Oct 24;22(10):1806. doi: 10.3390/molecules22101806 (PMC6151378; doi:10.3390/molecules22101806)
Supplement: Supplementary file 1 [file molecules-22-01806-s001.pdf]

Supplementary material

Enhanced glucose uptake in human liver cells and inhibition of carbohydrate hydrolyzing enzymes by Nordic berry extracts

Giang Thanh Thi Ho[a], Thi Kim Yen Nguyen[a,b], Eili Tranheim Kase[b], Margey Tadesse[a], Hilde Barsett[a] and Helle Wangensteen[a*]

[a] Department of Pharmaceutical Chemistry, School of Pharmacy, University of Oslo, P. O. Box 1068 Blindern, 0316 Oslo, Norway

[b] Department of Pharmaceutical Biosciences, School of Pharmacy, University of Oslo, P. O. Box 1068 Blindern, 0316 Oslo, Norway

Supplementary material: ^{1}H NMR spectra of SPE berry extracts used in this study.

S1. Bilberry SPE Extract

S2. Blackberry SPE Extract

S3. Black Chokeberry SPE Extract

S4. Black Currant SPE Extract

S5. Blueberry SPE Extract

S6. Bog Whortleberry SPE Extract

S7. Cloudberry SPE Extract

S8. Crowberry SPE Extract

S9. Elderberry SPE Extract

S10. Lingonberry SPE Extract

S11. Raspberry SPE Extract

S12. Red Currant SPE Extract

S13. Rowanberry SPE Extract

S14. Sea Buckthorn SPE Extract

S1.

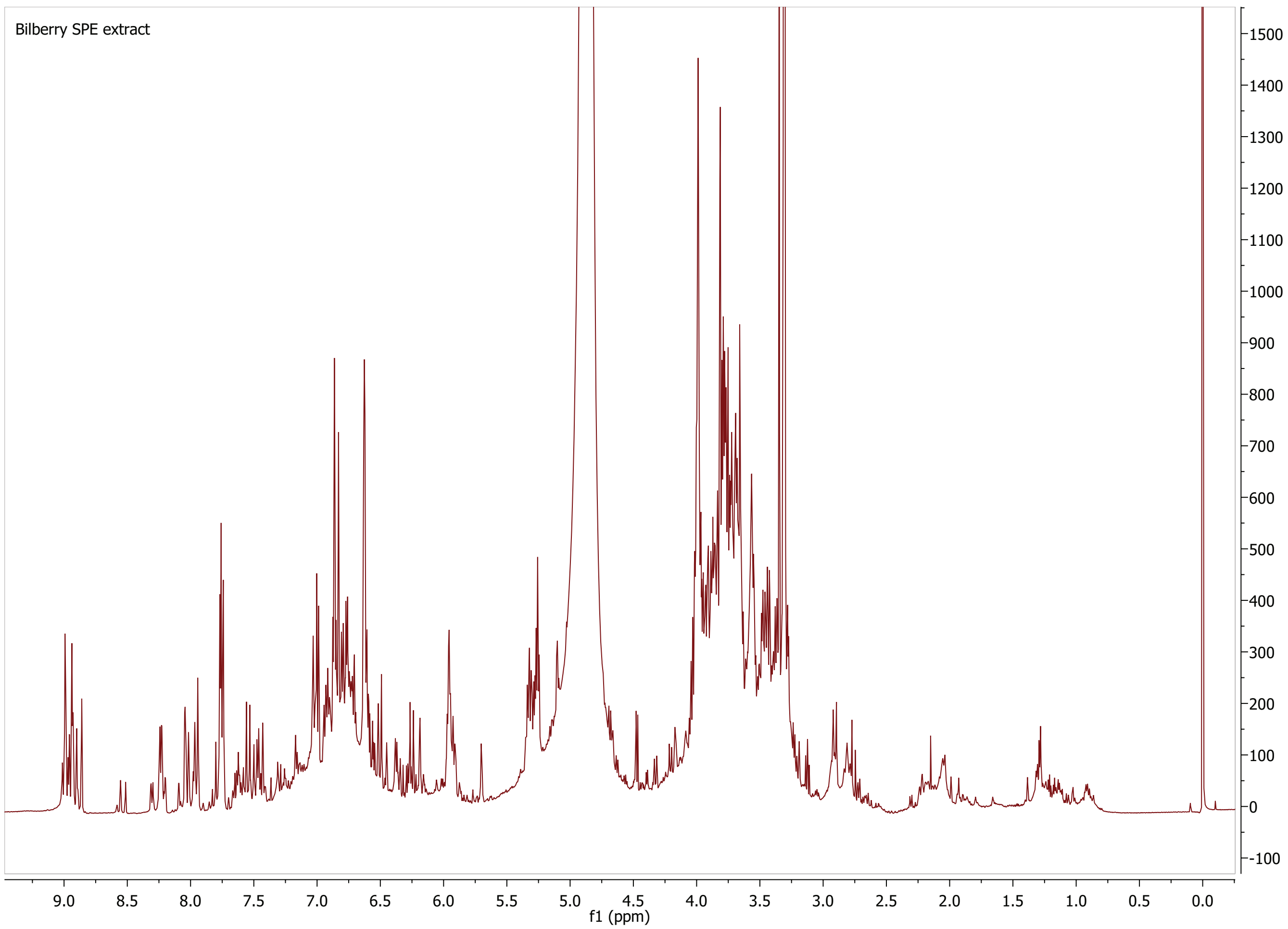

S2.

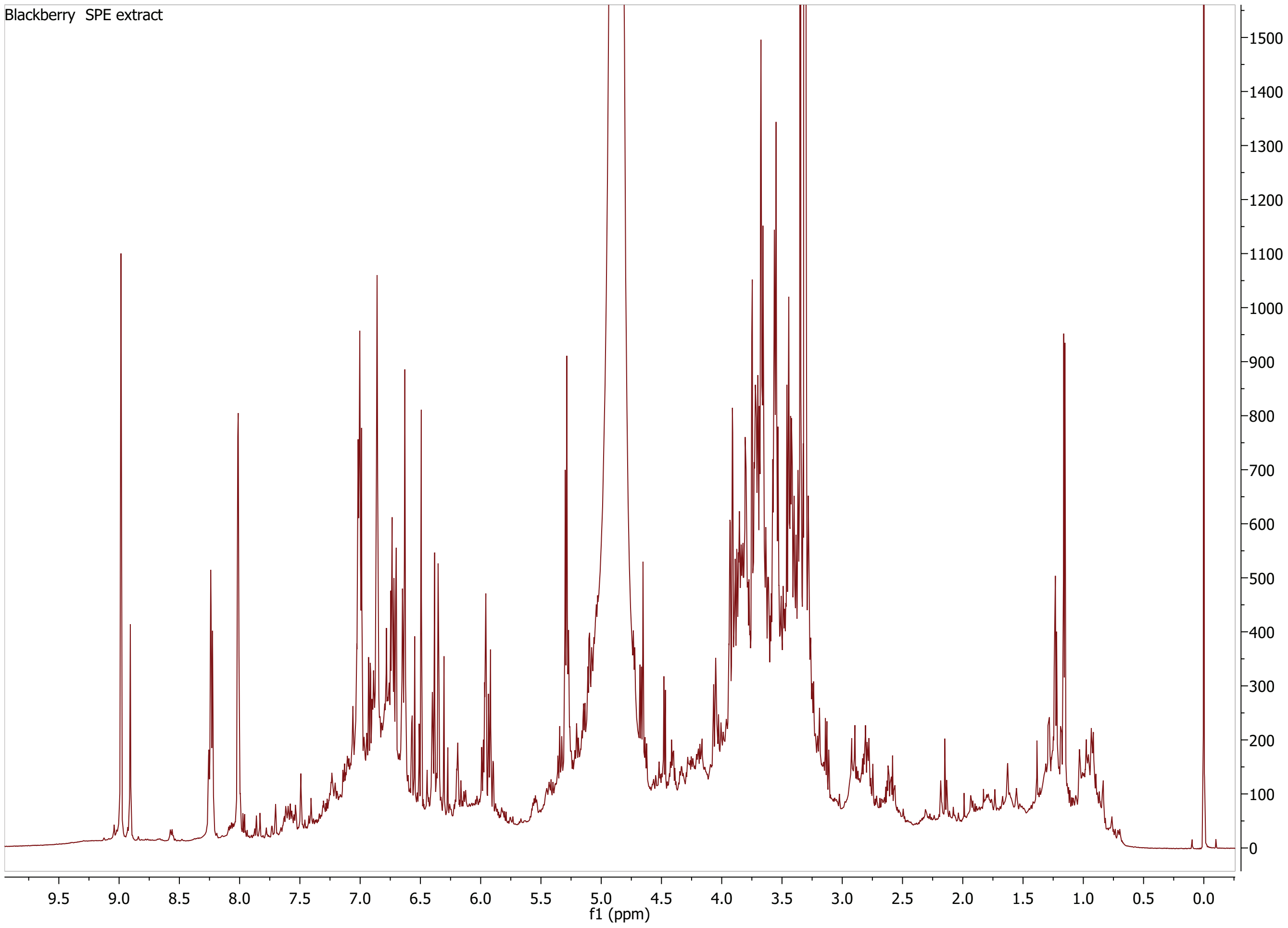

S3.

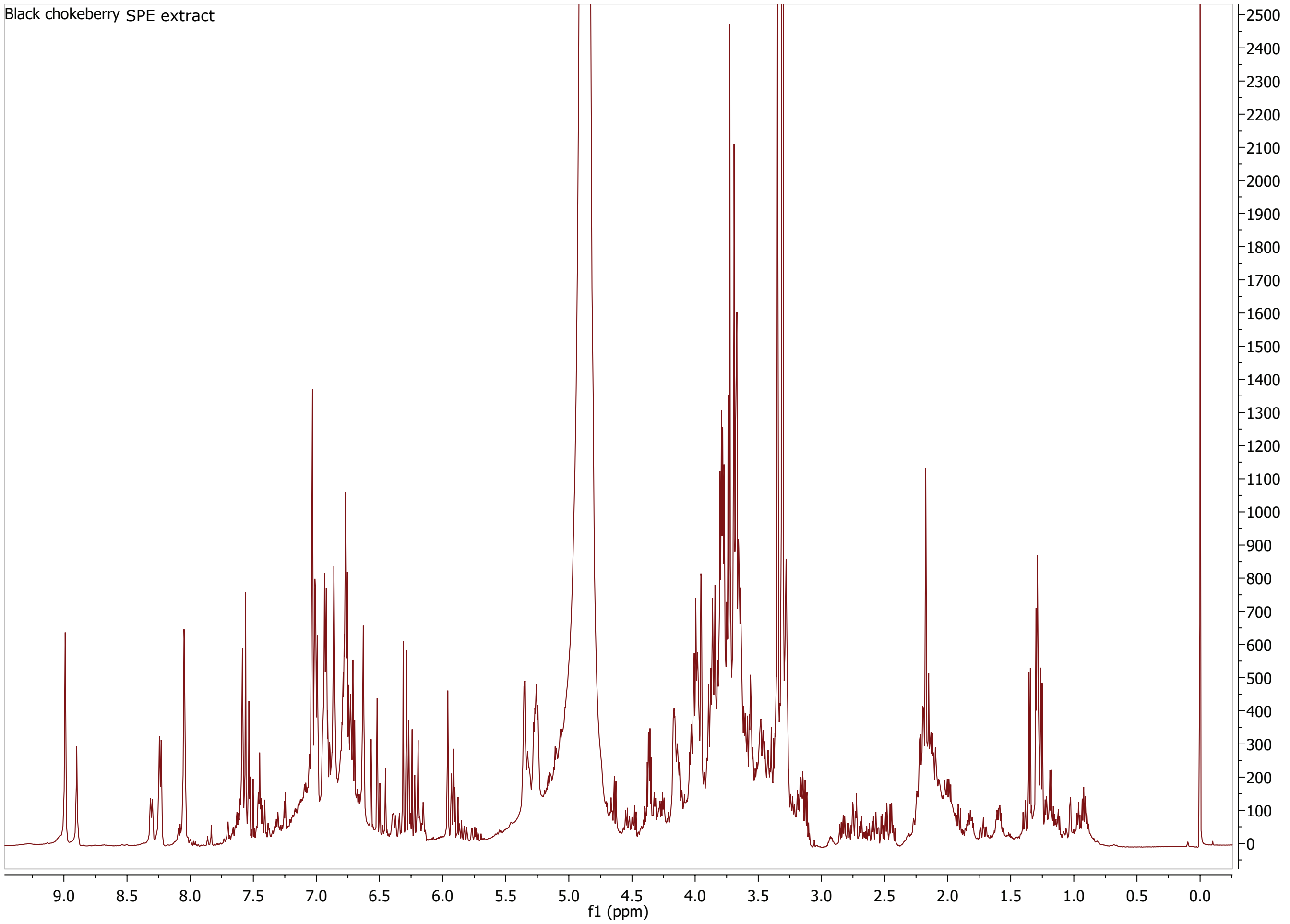

Black chokeberry SPE extract
2500
2400
2300
2200
2100
2000
1900
1800
1700
1600
1500
1400
1300
1200
1100
1000
900
800
700
600
500
400
300
200
100
0
9.0
8.5
8.0
7.5
7.0
6.5
6.0
5.5
5.0
4.5
4.0
3.5
3.0
2.5
2.0
1.5
1.0
0.5
0.0
f1 (ppm)

S4.

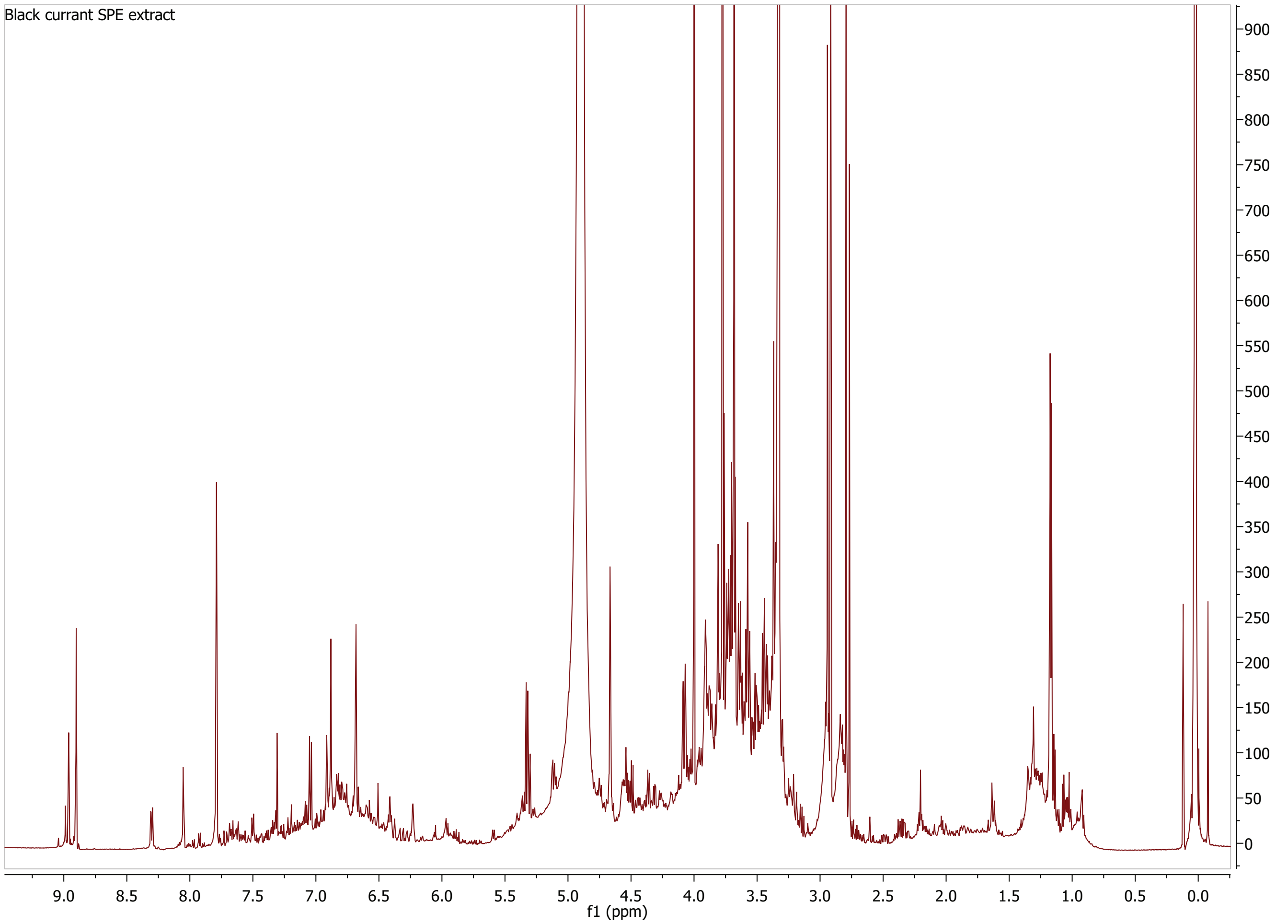

Black currant SPE extract
900
850
800
750
700
650
600
550
500
450
400
350
300
250
200
150
100
50
0
9.0
8.5
8.0
7.5
7.0
6.5
6.0
5.5
5.0
4.5
4.0
3.5
3.0
2.5
2.0
1.5
1.0
0.5
0.0
f1 (ppm)

S5.

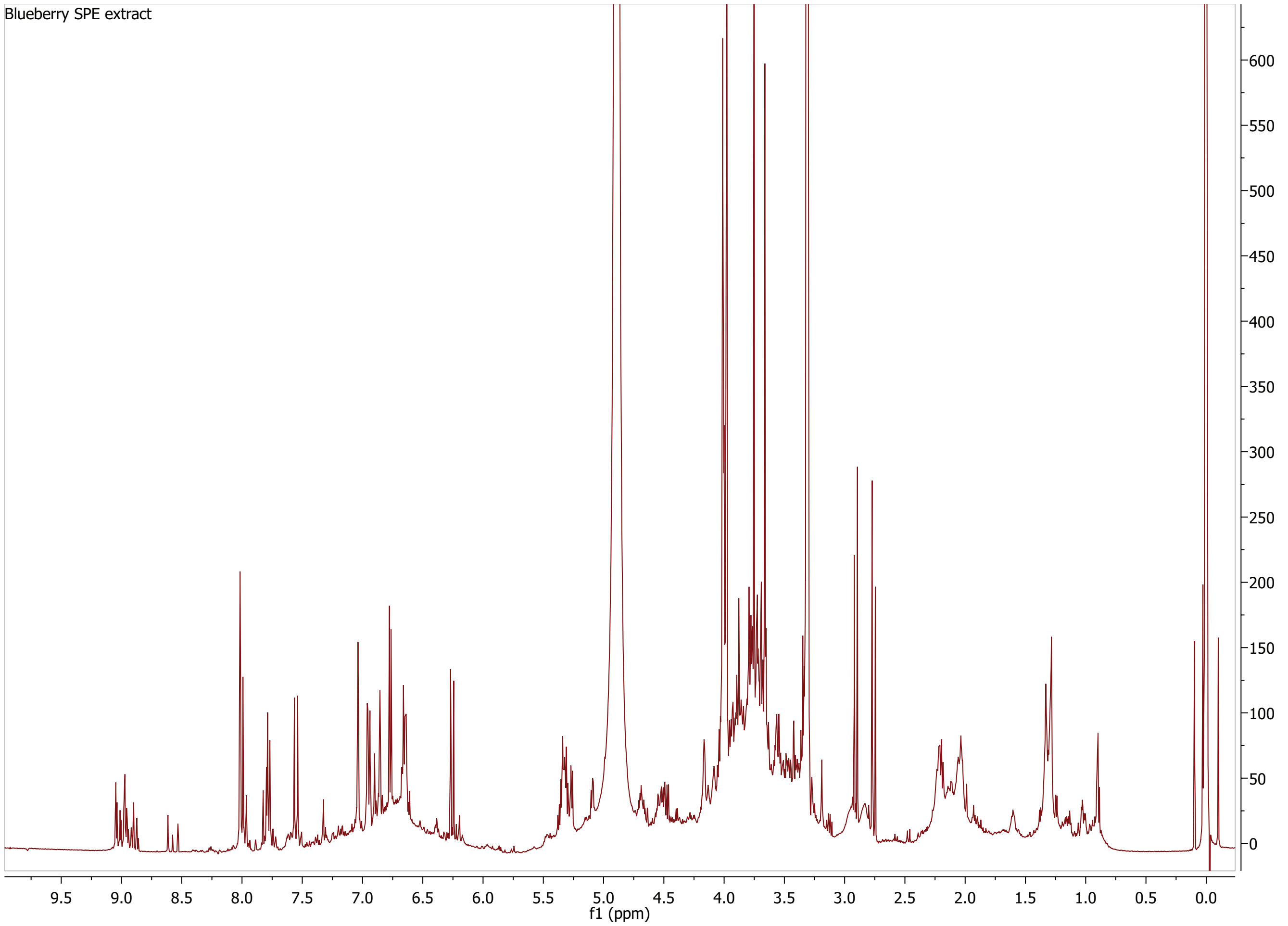

Blueberry SPE extract
f1 (ppm)

S6.

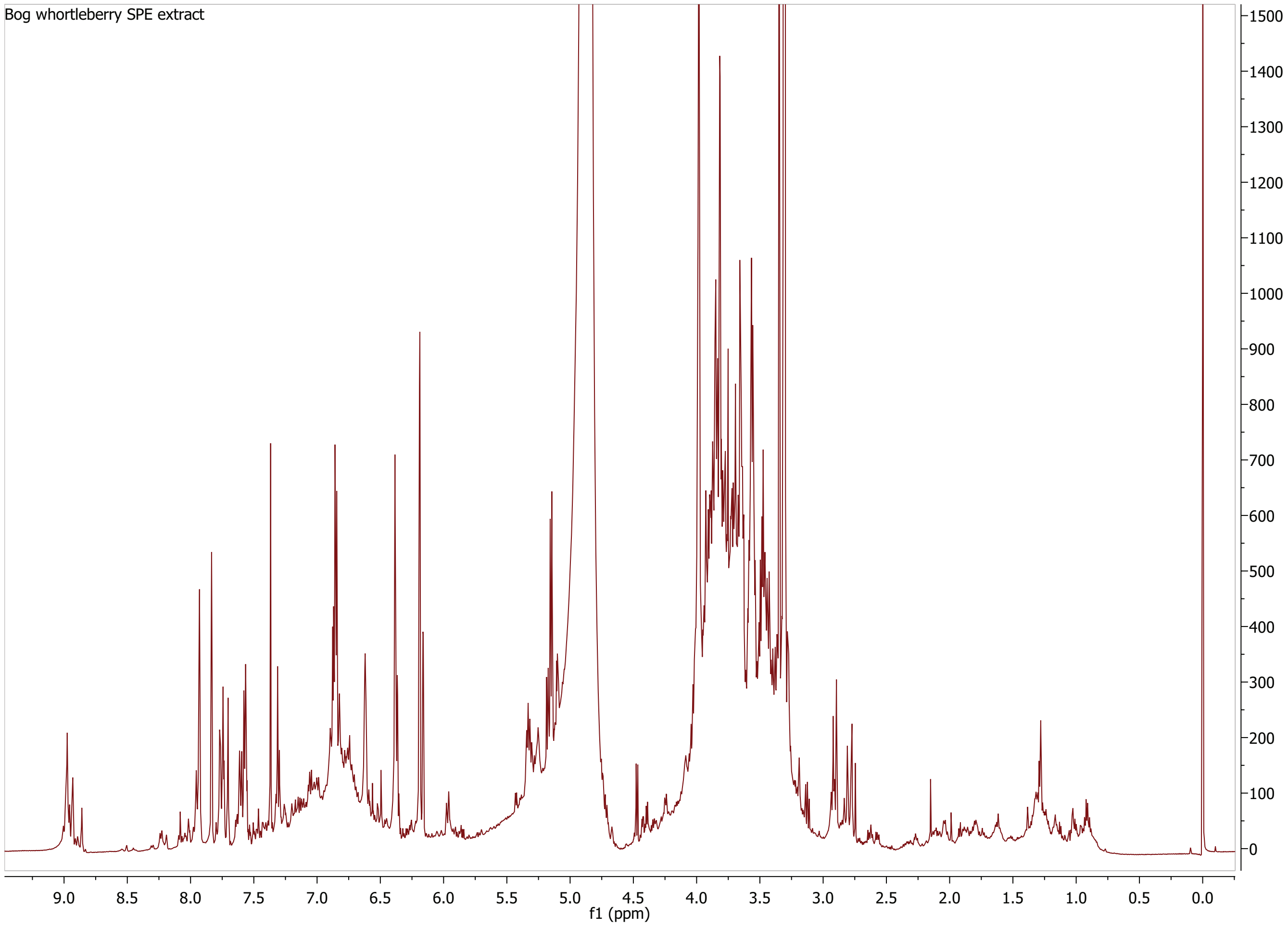

Bog whortleberry SPE extract
f1 (ppm)

S7.

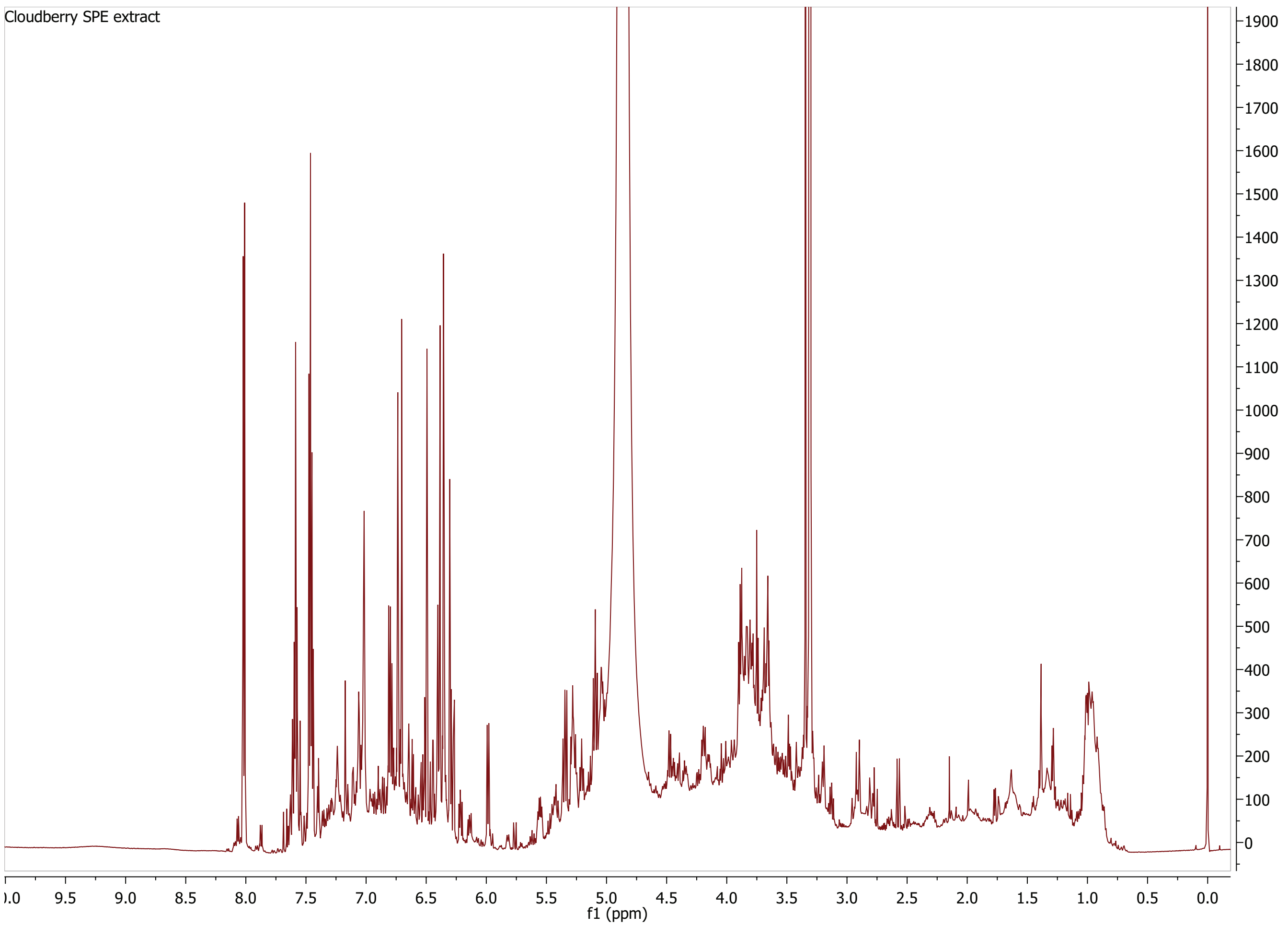

S8.

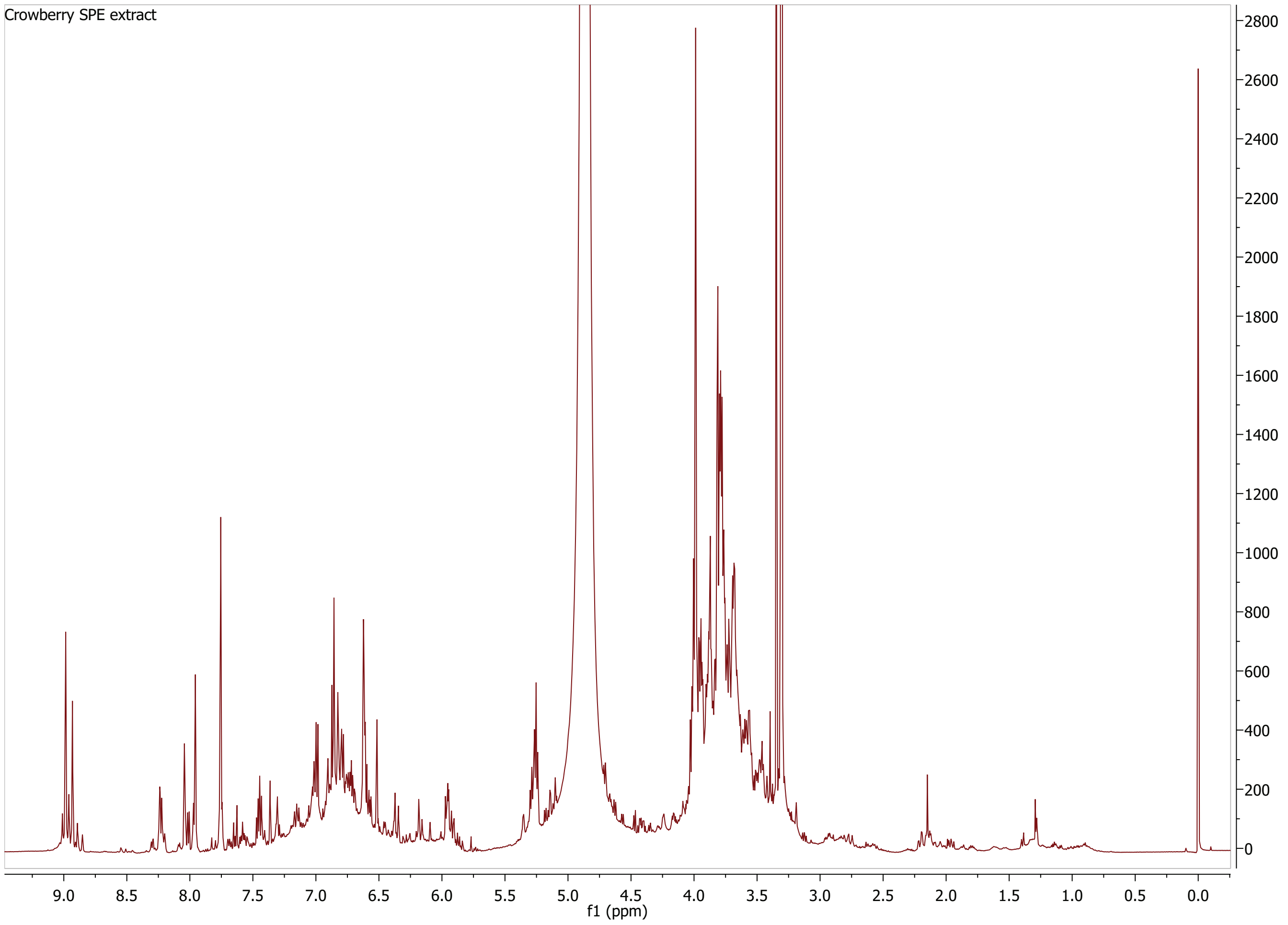

S9.

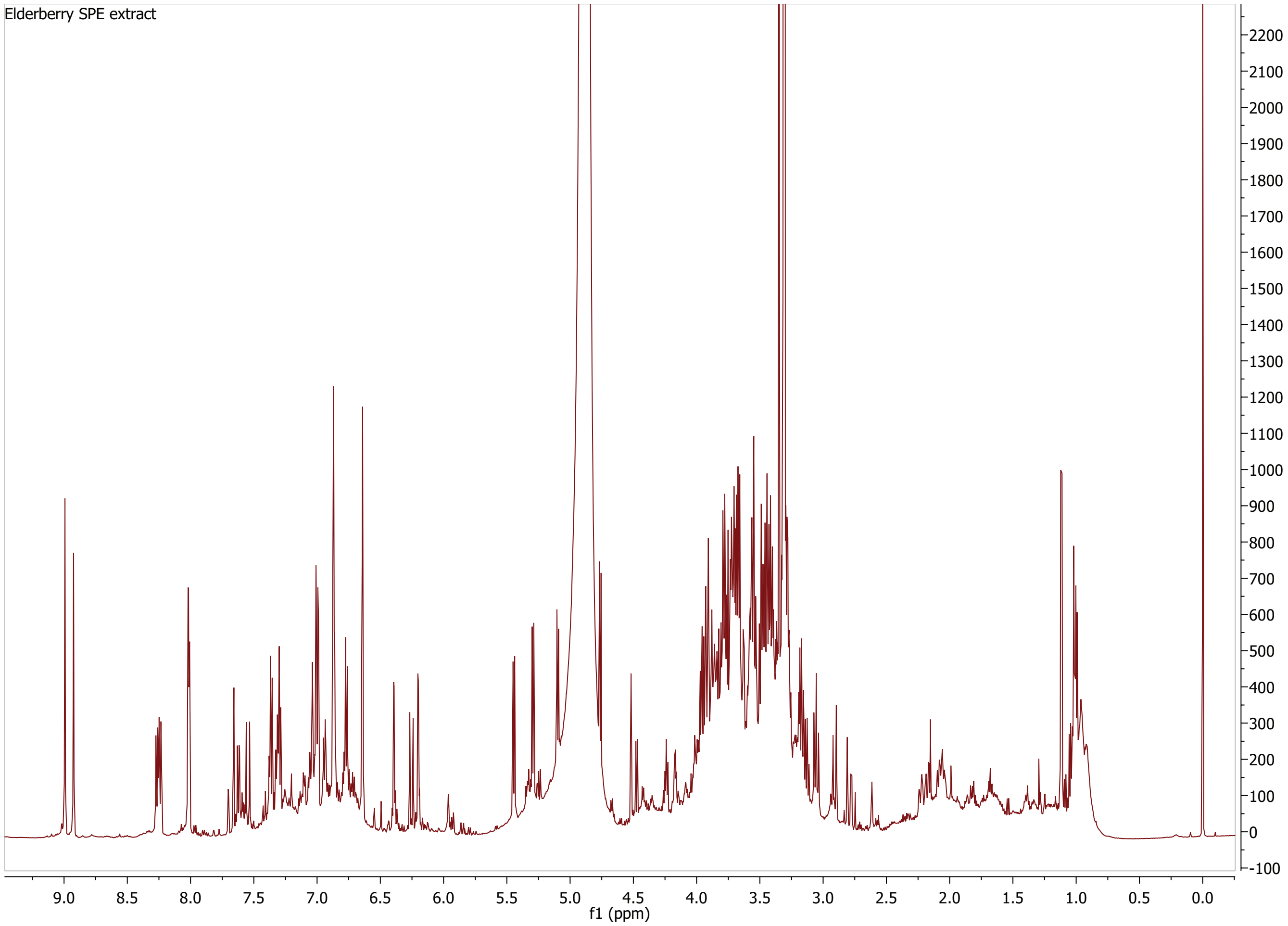

Elderberry SPE extract
2200
2100
2000
1900
1800
1700
1600
1500
1400
1300
1200
1100
1000
900
800
700
600
500
400
300
200
100
0
-100
9.0 8.5 8.0 7.5 7.0 6.5 6.0 5.5 5.0 4.5 4.0 3.5 3.0 2.5 2.0 1.5 1.0 0.5 0.0
f1 (ppm)

S10.

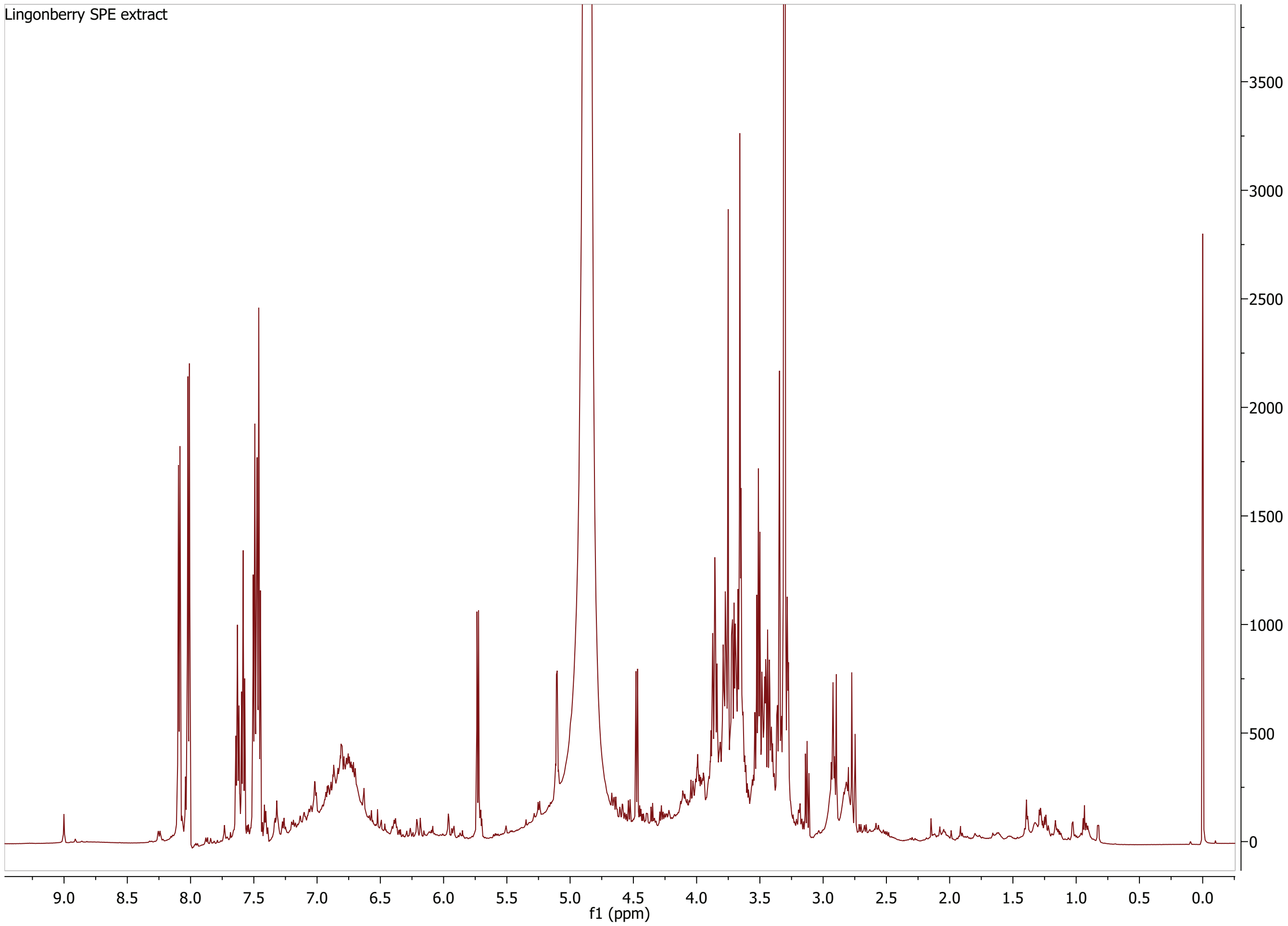

Lingonberry SPE extract
3500
3000
2500
2000
1500
1000
500
0
9.0 8.5 8.0 7.5 7.0 6.5 6.0 5.5 5.0 4.5 4.0 3.5 3.0 2.5 2.0 1.5 1.0 0.5 0.0
f1 (ppm)

S11.

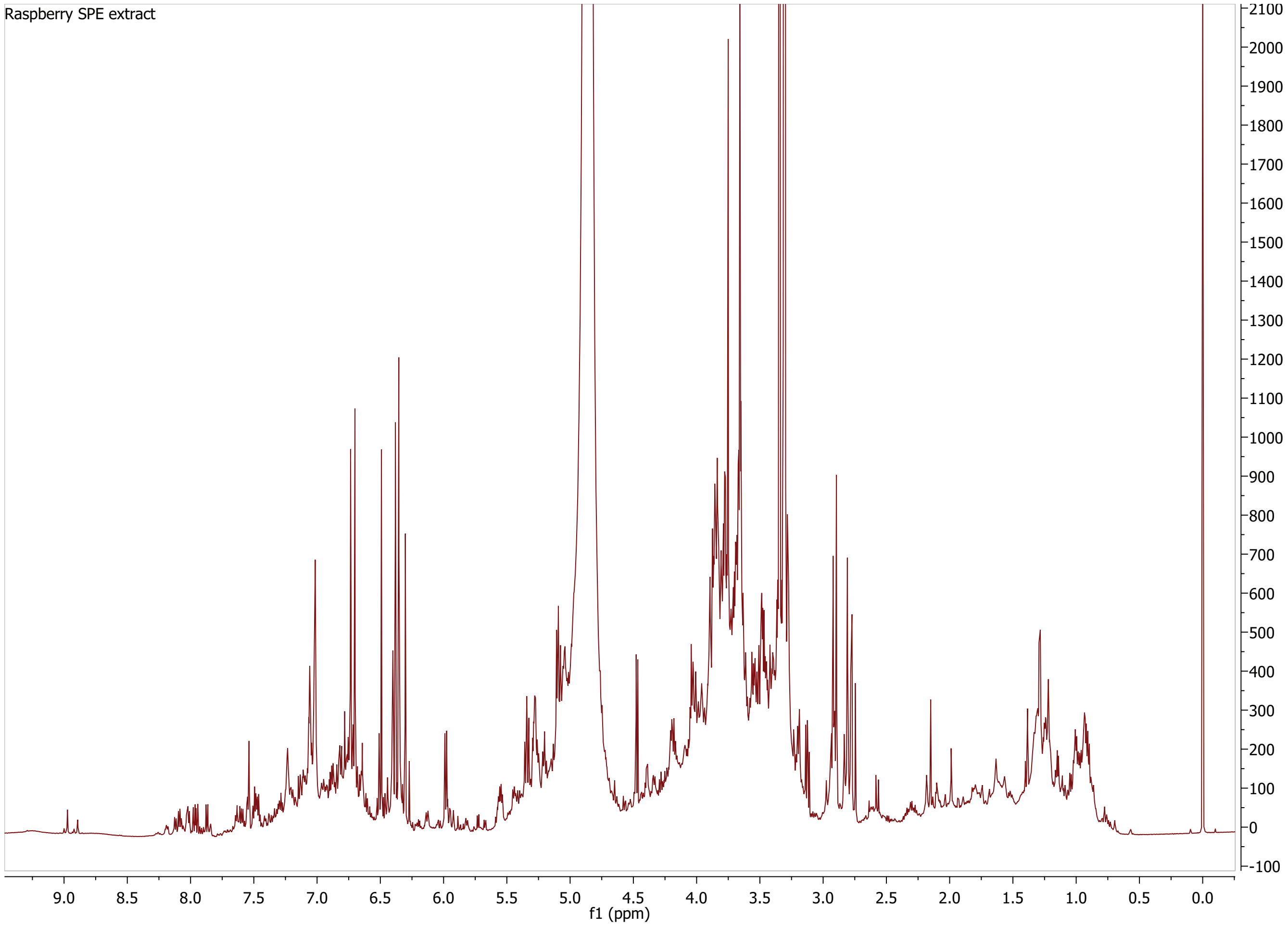

S12.

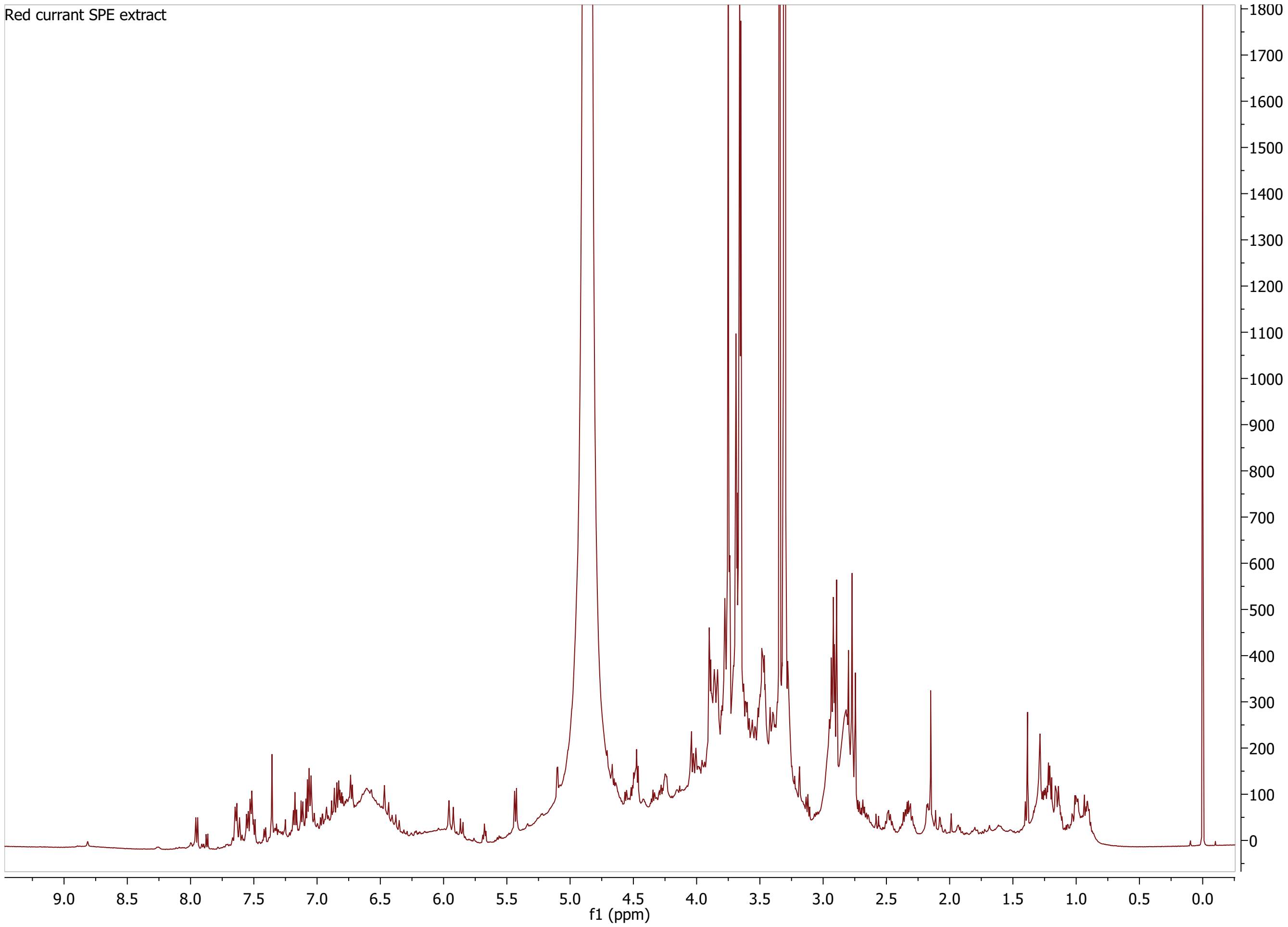

S13.

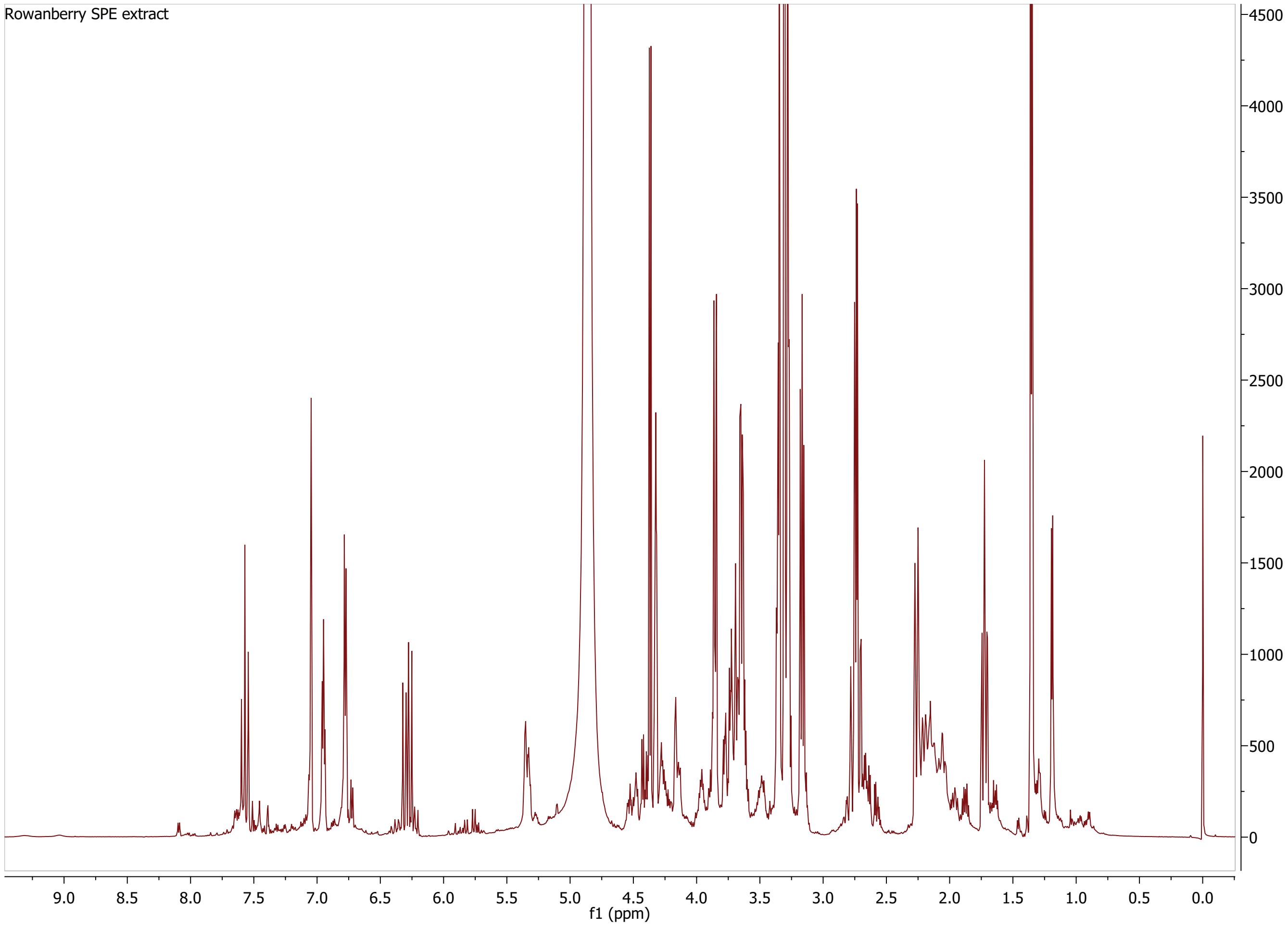
Rowanberry SPE extract
4500
4000
3500
3000
2500
2000
1500
1000
500
0
9.0
8.5
8.0
7.5
7.0
6.5
6.0
5.5
5.0
4.5
4.0
3.5
3.0
2.5
2.0
1.5
1.0
0.5
0.0
f1 (ppm)

S14.

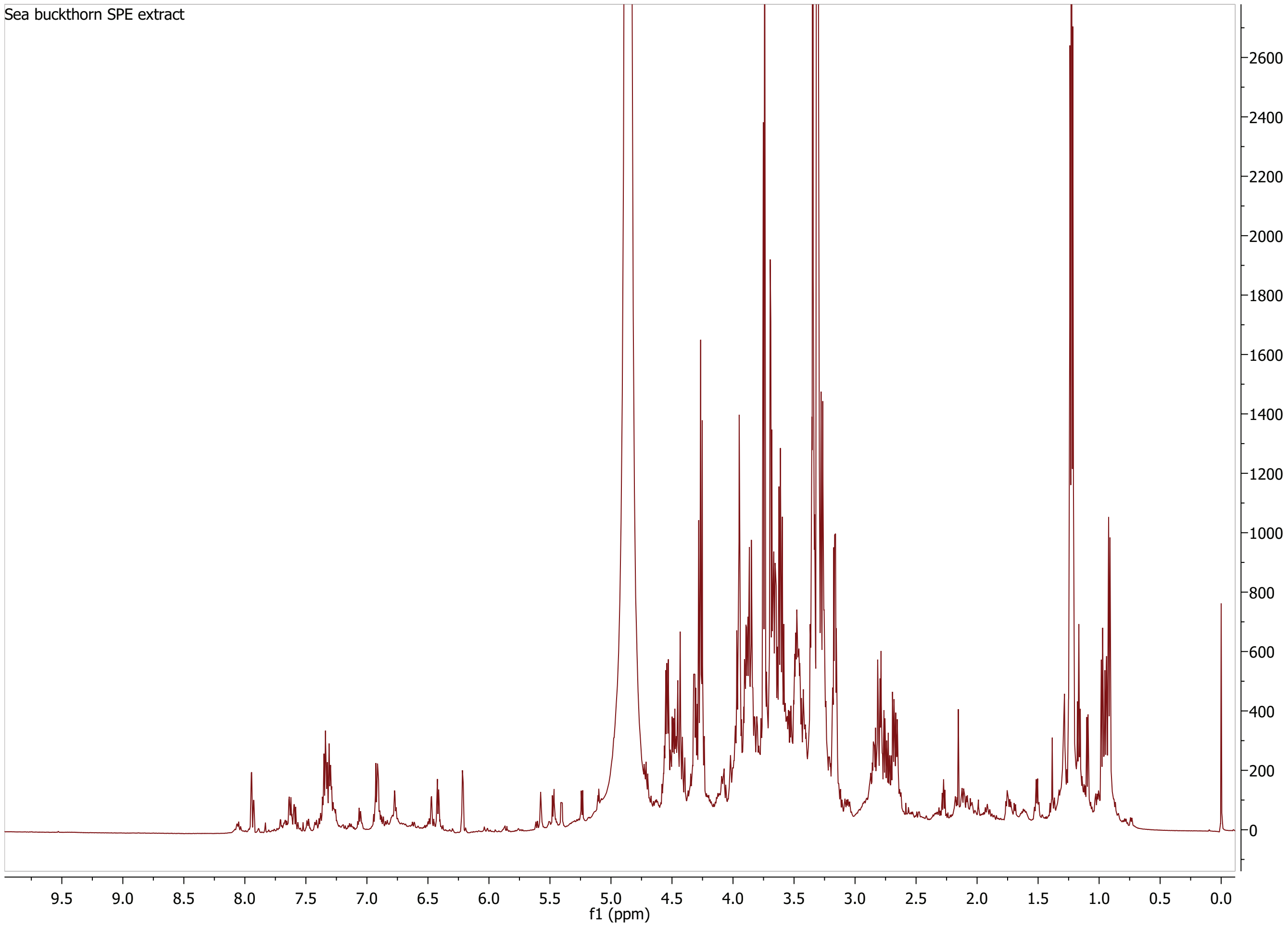
Sea buckthorn SPE extract
2600
2400
2200
2000
1800
1600
1400
1200
1000
800
600
400
200
0
9.5
9.0
8.5
8.0
7.5
7.0
6.5
6.0
5.5
5.0
4.5
4.0
3.5
3.0
2.5
2.0
1.5
1.0
0.5
0.0
f1 (ppm)
